# A Piezoresistive Sensor with High Sensitivity and Flexibility Based on Porous Sponge

**DOI:** 10.3390/nano12213833

**Published:** 2022-10-30

**Authors:** Hengyi Yuan, Yi Li, Zhihui Qian, Lei Ren, Luquan Ren

**Affiliations:** 1 Key Laboratory of Bionic Engineering, Jilin University, Changchun 130022, China; 2 School of Mechanical and Vehicle Engineering, Jilin Engineering Normal University, Changchun 130052, China; 3 School of Mechanical, Aerospace and Civil Engineering, University of Manchester, Manchester M13 9PL, UK

**Keywords:** flexible piezoresistive sensor, porous structure, high sensitivity, electroless plating process

## Abstract

Chemical plating has recently been employed for the preparation of flexible piezoresistive sensors; however, plating solutions and processes that affect the sensitivity still need further exploration. In the study, a sponge-based flexible sensor with copper as its conductive material is prepared using electroless plating. The variation in sponge resistance and sensitivity changes with different plating times are studied. It is found that, with the increasing plating time, the conductivity increases and the resistance of sample will decrease. Moreover, the range of resistance difference will decrease under compression, thus the sensitivity decreases. Furthermore, the sensor’s applications were assessed, verifying the practicability of the developed preparation method. This study may bring ideas for the new development of flexible pressure sensors.

## 1. Introduction

In recent years, with the development of wearable gadgets used in health monitoring, research on sensors has greatly deepened and expanded, with aims of high quality, flexibility, sensitivity, and response speed [1,2,3,4,5]. Flexible sensors can easily attach to individuals’ skin, solving the mismatch between rigid planar sensors and human body curves. Based on their working mechanism, flexible sensors are divided into capacitive, piezoelectric, and piezoresistive sensors used for monitoring pressure, temperature, humidity, and so on [6,7,8,9,10,11].

Flexible piezoresistive sensors have attracted significant attention and are widely used in electronic skin, soft robotics, health monitoring, and so on [12,13]. There were many attempts in the applied magnetism and engineering to create flexible pressure sensors of different types. Melzer fabricated [Co/Cu] multilayers revealing a giant magnetoresistance (GMR) effect on free-standing elastic poly (dimethylsiloxane) (PDMS) membranes [14]. A series of multilayer thin-film structures based on Fe20Ni80 and Fe11Ni89 with Cu or Ta spacers were designed, prepared, and investigated [15]. Flexible magnetoimpedance (MI) sensors fabricated using a NiFe/Cu/NiFe tri-layer on Kapton substrate have been studied by Li [16]. Many methods have been developed for preparing high-performance flexible piezoresistive sensors [17,18,19]. Conductive nanomaterials, such as graphene, carbon nanotubes, and metal nanomaterials, with elastomeric material, such as PDMS, PU, SBS, and Ecoflex, are integrated to form composites [20,21,22,23,24,25,26,27,28]; however, for the sensors to perform well, conductive fillers and elastomeric materials must be controlled. For example, the composite conductive thin film was prepared with a spraying method by shadow masks [29]. This preparation method is simple and low-cost, but the electrical property is largely affected by material deformation and the sliding between conductive nanomaterials. To solve the problems, the sensor structure is designed with regard to the wave structure, effectively decreasing the influence of material deformation [30,31]. Other sensor structures, such as spring and net-shaped, have been developed [32,33,34]. Although the preparation methods, such as template and electrospinning, coupled with the designed sensor structures have further applications [35,36], the requirements of material stretchability and stability are still difficult to meet, along with mass production challenges [37].

In recent years, materials with three-dimensional (3D) network structures have been widely used in every field thanks to their simple and economical production along with excellent stretchability and stability [38,39,40]. Graphene with 3D networks has been extensively studied for its great electrical conductivity and mechanical performance. Nevertheless, some preparation methods, such as CVD and hydrothermal reduction, are complicated [41,42,43,44]. For instance, three-dimensional porous polymer composites with graphene networks prepared by CVD and PEDOT/PSS coating showed high conductivity [38]. However, their complicated and costly preparation methods make mass production and wide application difficult. In a recent report, 3D graphene sponge flexible sensors with great performance are prepared by the electrodeposition method, but the derived microcracks significantly influence the resistance variations when stretched [45]. In further research, the chemical plating method is applied in the preparation of 3D graphene sponge flexible sensors; compared with the electrode position method, there are no microcracks in the preparation process, making it suitable for good conductivity and uniform coating. Although chemical plating is appropriate for preparing flexible piezoresistive sensors, plating solutions and other experimental processes affect the sensitivity [46], requiring further exploration.

In this paper, the chemical plating method is applied for preparing high-sensitivity and flexible piezoresistive sensors based on the porous sponge, the experiment cost is low, and finite element analysis is employed to demonstrate the flexible piezoresistive sensors’ mechanism. The sensitivity is raised to 98.5% and the application of wearable gadgets is verified by flexible circuit.

## 2. Materials and Methods

### 2.1. Materials

Materials: Sponge (polyurethane, 5 mm thickness) was produced by Nanjing Saneduo Sponge (Nanjing, China); CuSO_4_ 5H_2_O (99 wt%) and HCl (37.5 wt%) were purchased from Beijing Chemical Works (Beijing, China); NaKC_4_H_4_O_6_ (99 wt%), Na_3_PO_4_·12H_2_O (98 wt%), SDBS (90 wt%), and Na_2_CO_3_ (99.8 wt%) were from Tianjin Guangfu Technology Development Co., Ltd. (Tianjin, China); NaOH (96 wt%) was from Tianjin Beichen Founder Reagent Factory (Tianjin, China; NiCl_2_·6H_2_O (98 wt%) and SnCl_2_·2H_2_O (98 wt%) were from Tianjin Yongsheng Fine Chemicals Co., Ltd. (Tianjin, China); HCHO (36 wt%) was from Liaoning Quan Rui Reagent Co., Ltd. (Liaoning, China); Op-10 emulsifier (99 wt%) was from Tianjin Zhiyuan Chemical Reagent Co., Ltd. (Tianjin, China); and PdCl_2_ (99.9 wt%) was from Tianjin Chemical Reagent Third Factory (Tianjin, China).

### 2.2. Preparation Methods

**Figure 1:**
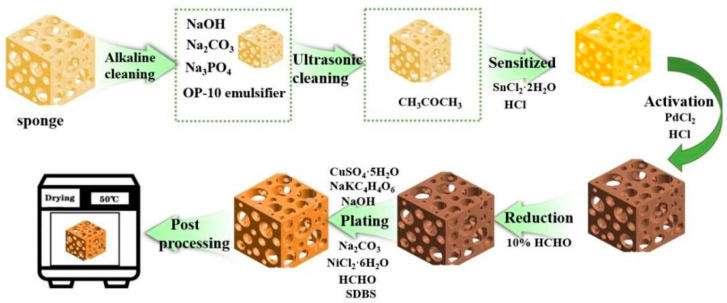
Schematic diagram of the flexible piezoresistive sensor preparation process.

A 5 mm thick low-density sponge was cut to 70 mm × 50 mm. There are no special requirements for the type and size of sponges. The types of sponges are common in the market. Other types of sponges are also operable. Sponge size is also important for the purpose of uniform specifications, and different sizes of sponges also have this feature. An electroless copper plating sponge composite mainly includes six steps: cleaning, sensitization, activation, reduction, plating, and post-treatment, as shown in Figure 1.

① Cleaning

The purpose of the substrate and, consequently, the sponge cleaning is to ensure obtaining the ideal catalytic activity, mainly including alkaline and ultrasonic cleaning.

A. Alkaline cleaning. Ingredients of alkaline cleaning solution: 20–40 g/L NaOH, 20–30 g/L Na_2_CO_3_, 5–10 g/L Na_3_PO_4_·12H_2_O, and 1–3 g/L OP-10 emulsifier. Alkaline cleaning process: 10 min at 70 °C.

B. Ultrasonic cleaning. After alkaline cleaning, the sample is immersed in CH_3_COCH_3_ solution, followed by ultrasonic cleaning for 2 min with an ultrasonic instrument, and then immersed in deionized water for 1 min.

② Sensitization

The purpose of the sensitization is to adsorb a layer of easily oxidized material inside and on the sponge’s surface as a reducing agent for catalytic metal ions during the subsequent activation treatment. The sensitizing solution comprises 20 g/L SnCl_2_·2H_2_O and 40 mL/L HCl. The sensitization process is carried out at room temperature for 6 min. During the sensitization process, the sponge must be pressed to ensure the sensitization liquid is fully in contact with the inner space support of the sponge. Finally, it is cleaned with a deionized water press for 30 s.

③ Activation

The purpose of activation is to generate a catalytic metal layer inside and outside of the sponge as a catalyst for the REDOX reaction during the following electroless plating. The activation solution comprises 1 g/L PdCl_2_ and 10 mL/L HCl. The activation process takes 6 min at room temperature and requires the sponge to be pressed to ensure that the activation fluid can make full contact with the inner space of the sponge. Finally, it is cleaned with a deionized water press for 30 s.

④ Reduction

The purpose of reduction is to improve the catalytic activity of the substrate’s surface, accelerate the deposition rate of the electroless copper plating solution, and prevent contamination of the electroless copper plating solution. The reducing solution comprises 10% HCHO. The reduction process involves stirring at room temperature for 3 min. During the reduction process, the sponge must be pressed to ensure the sensitization liquid is fully in contact with the inner space support of the sponge.

⑤ Plating

The purpose of plating is to reduce the metal ions in the electroless plating solution to their metal state and deposit them on the surface of the plated parts to form a copper film. The plating solution consists of 10 g/L CuSO_4_·5H_2_O, 40 g/L NaKC_4_H_4_O_6_, 8 g/L NaOH, 2 g/L Na_2_CO_3_, 1 g/L NiCl_2_·6H_2_O, 20 mL/L HCHO(36%), and 0.5 g/L SDBS. The plating process takes 30 s of being pressured in a 40 °C water bath box.

⑥ Post-processing

The purpose of post-treatment is to remove the residual liquid and means processing in a drying oven at 50 °C for 30 min after thoroughly cleaning with deionized water.

### 2.3. Testing Methods

Scanning electron microscopy (SEM, SU3500, HITACHI, Tokyo, Japan) was employed to observe the samples’ morphologies. The accelerating voltage of the SEM microscope was 15 kV, measuring the distance at 58 mm, and the magnification was 50.

An energy-dispersive spectrometer (EDS, 550i, IXRF, Austin, TX, USA) was used to determine the samples’ elemental composition. The EDS’s accelerating voltage was 15 kV, with a take-off angle of 35.0°.

**Figure 2:**
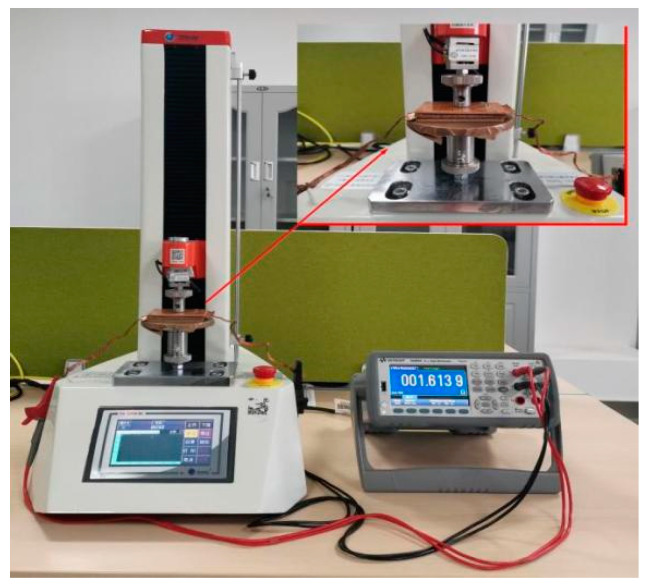
Sensor performance test system ((**left**): press, (**right**): multimeter, (**inset**): resistance measuring point).

A universal testing machine (ZQ-990B, Dongguan Zhigu Precision Instrument Co., Ltd., Dongguan, Guangdong, China) and a digital multimeter (KEYSIGHT-34465A, KEYSIGHT Technologies, Springs, CO, USA) (as shown in Figure 2) were used to analyze the performance of the attained sensor. During the measurement, two pieces of copper foil were placed on the upper and lower surfaces of the sensor to act as conductors and the measuring surface (as shown in the inset of Figure 2). As the indenter moves up and down, it can better fit the surface of the sample, which is more accurate than the point measurement. Here, 0–100 N force was dynamically applied, with the pressure speed set as 1 mm/min. The press exerts a force of 0 N–100 N on the sponge during the compression process, gradually compressing and deforming the sponge and thinning the pores. When the loading force reaches 100 N, the compression process stops. Under the deformation state of the 100 N force applied by the press, it gradually decreases, and the sponge’s pores recover during the recovery process. When the pressing force on the sponge measures at zero N, the sponge recovers partial deformation before the cyclic compression. The press repeatedly applies a force of 0–100 N, taking 20 cycles as an example.

### 2.4. Finite Element Analysis

Finite element analysis was employed to analyze the performance improvement mechanism of the attained flexible sensor. The finite element software ANSYS (Version 2021, ANSYS, Canonsburg, PA, USA) was used to simulate the feasibility of the material as a flexible sensor. The sponge compression process was simplified as only one porous material and the press was simplified as an applied displacement. In the static analysis, the density of the sponge is 40 kg/m^3^, Poisson’s ratio is 0.38, and the modulus of elasticity is 0.9 Gpa; the material was assumed as a flexible body owing to its deformation under pressure. The porous material is meshed using 36,073 tetrahedral elements, determined through a convergence analysis by gradually increasing the mesh density until the deviations in the estimated stress reach <5%. In order to control the sponge’s deformation accurately, displacement was applied at the top of the material and fixed constraints were applied at the bottom. The finite element analysis was divided into two steps. At first, the flexible material was not deformed, followed by 5%, 10%, 15%, 20%, and 25% deformations. Secondly, a 12 V voltage to the top of the undeformed and deformed material and a 0 V voltage at the bottom were applied, obtaining the resistance variation trends by averaging the current density at the bottom of the material.

## 3. Results and Discussions

### 3.1. Structural Analysis

In this study, electroless plating was used for the first time to attach the copper to the sponge’s surface and to its pores to functionalize the material. Many studies ignored the high possibility of nanoparticles separating from the matrix material and causing severe health problems to users when used as a wearable gadget for motion detection; therefore, this study used harmless copper as the conductive filler material of the sponge.

**Figure 3:**
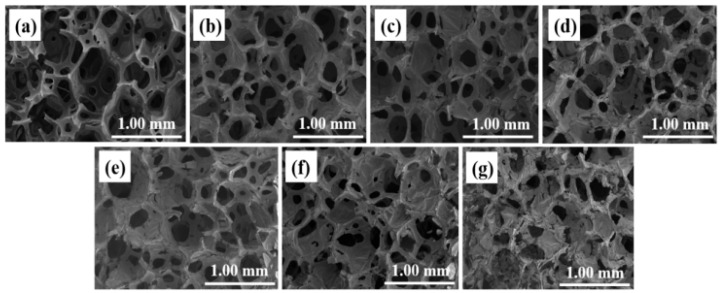
Scanning electron microscopy images of samples with different plating times: (**a**) unplated, (**b**) 1 min, (**c**) 1.5 min, (**d**) 2 min, (**e**) 4 min, (**f**) 8 min, and (**g**) 10 min.

Figure 3 shows the SEM images of sponges with different plating times. Figure 3a shows the SEM images of sponges without copper plating. As can be seen from the sample cross section, the sponge has a network structure inside without any attachments. Figure 3b–g show samples with different plating times ranging from 1 to 10 min. It can be deduced that the sample’s surface changes and the sponge is covered with a layer of dense copper with folds evenly distributed. The copper film uniformly covers the surface and the sponge’s pores and forms a network. The sponge’s pores fill up with the increasing plating time and the copper film of the sample thickens, demonstrating that copper is successfully deposited on the sponge’s surface. The reduced copper is coated on the sponge during preparation and penetrates the interconnecting holes. The SEM images show the microstructure of the copper-coated sponge and the interconnecting pores covered by the copper.

### 3.2. Elemental Analysis

**Figure 4:**
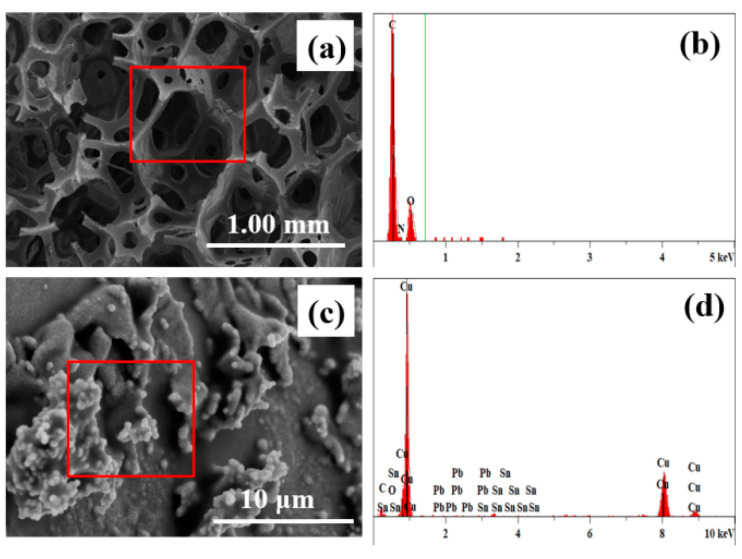
(**a**) The SEM and (**b**) energy spectrum of the sample before copper plating; (**c**) the SEM and (**d**) energy spectrum of the sample after copper plating.

Based on the electroless plating method, the copper mass fraction in the sponge’s pores is controlled by the plating time in the study. Figure 4 shows the energy spectrum of sponge samples before and after plating. As shown, the elements in the sample without plating are mainly carbon and oxygen, with the carbon content being the largest. After plating, a tiny amount of palladium, tin, and a large amount of copper appeared, while the proportion of copper increased significantly to about 95%.

### 3.3. Mechanical Property Analysis

**Figure 5:**
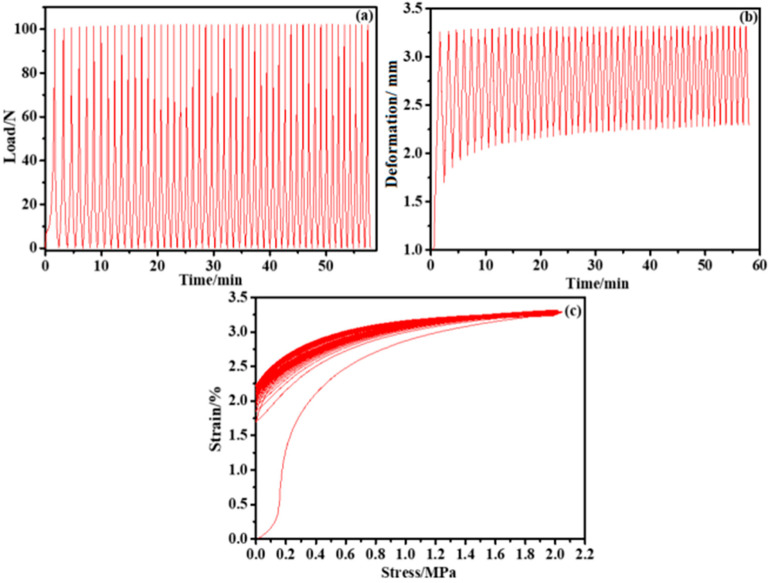
(**a**) Cyclic force curve applied by the press, (**b**) cyclic compression deformation curve of the sample, and (**c**) the stress–strain curve of the sample.

Sponge is a kind of material with a porous structure; it is insulated, soft, elastic, and does not easily undergo plastic deformation. Based on the above characteristics, the sponge is analyzed after copper plating. Figure 5 shows the deformation of the sponge-based sensor subjected to cyclic compression load, divided into two phases of compression and recovery. Figure 5a,b show the load and sponge’s deformation during the first hour of testing. The load is cycled with the test time changes. When the maximum peak value force (100 N) is applied, the deformation of the sponge is the largest, and it is the least when applying the minimum peak value (0 N). Increasing the test time brings the sponge deformation closer to stability at the tenth cycle (at minute 15). Figure 5c shows the stress–strain curve, demonstrating that the sponge can still be nearly restored to its initial state under many load cycles, thus indicating that the compressibility of a sponge-based material is a feasible flexible sensor.

### 3.4. Finite Element Analysis

**Figure 6:**
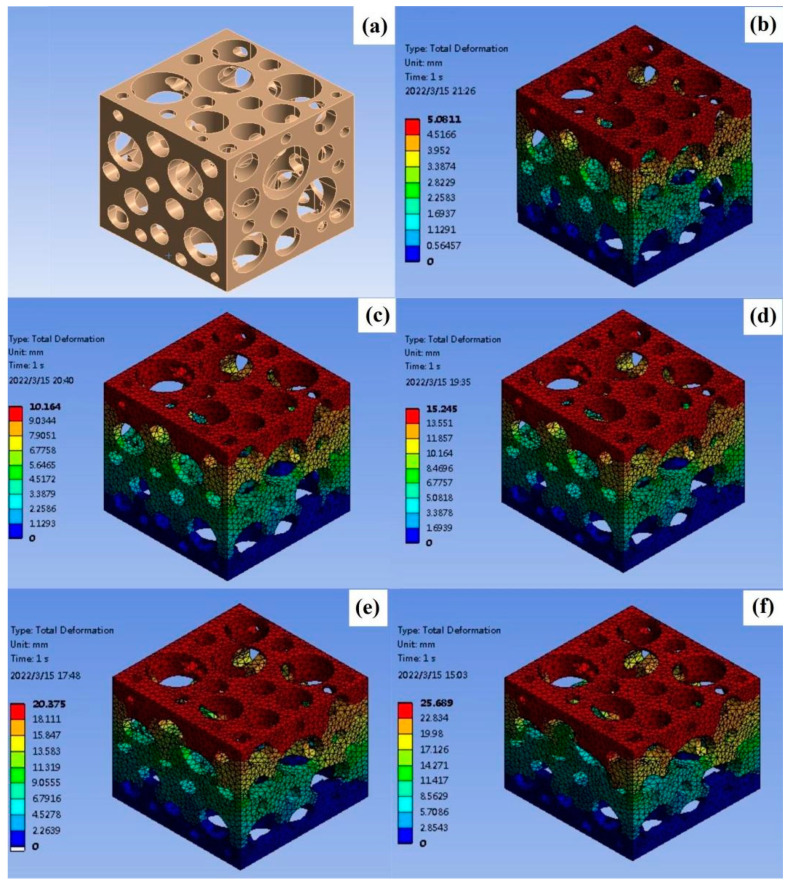
Deformation of sponge with different displacement constraints: (**a**) no deformation, (**b**) 5%, (**c**) 10%, (**d**) 15%, (**e**) 20%, and (**f**) 25%.

**Figure 7:**
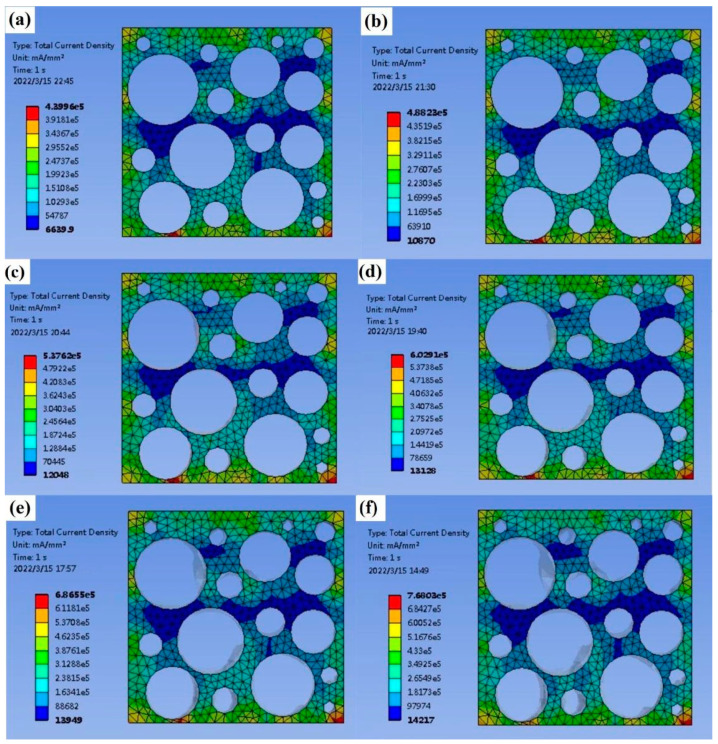
Current density of sponges with different deformations: (**a**) no deformation, (**b**) 5%, (**c**) 10%, (**d**) 15%, (**e**) 20%, and (**f**) 25%.

As shown in Figure 6 and Figure 7, in the finite element model, the variation law of resistance can be obtained by measuring the current/current density at the same voltage. The voltage of the upper surface of the sample is set to 12 V, the voltage of the lower surface is set to 0 V, and the meshes are unchanged. The average current density is obtained by solving the model. The average current density of the undeformed sponge is 142,260 mA/mm^2^, the average current density of 5% deformation is 176,070 mA/mm^2^, the average current density of 10% deformation is 192,460 mA/mm^2^, the average current density of 15% deformation is 208,800 mA/mm^2^, the average current density of 20% deformation is 223,320 mA/mm^2^, and the average current density of 25% deformation is 230,590 mA/mm^2^. It can be seen that increasing the deformation increases the current density and decreases the resistance against a constant voltage. Among the prepared materials, the copper-coated sponge has relatively high conductivity. This is because of the fact that a large amount of deformation under compression will directly increase the effective cross-section area and decrease the length of the sponge pore unit, thus decreasing the electrical resistance, leading to the resistance variations according to different deformations. In addition, the deposition thickness of copper increases with the increasing plating time within the study (as shown in Figure 3), indicating the copper content deposited in sponge pore is improved. Under this condition, the electrical resistivity will decrease, and it can be inferred the copper-coated sponge has better conductivity. However, in this study, when the reaction time is too long, the prepared plating solution will be out of work at the temperature of the water bath, so this effect is not observed with a long plating time. Therefore, the copper-coated sponge has advantages to be selected as a flexible piezoresistive sensor material.

### 3.5. Sensor Performance Analysis

The process used in this study can produce sensors with different sensitivities. Therefore, it is necessary to characterize and compare their sensitivities by various methods to determine their performance, benefits, and potential applications. Through the electroless plating of copper on the surface of the sponge, it can conduct electricity. The copper-plated sponge is equivalent to a flexible sensor and shows good electrical conductivity. ∆R/R_0_ represents the sensitivity, where ∆R = R_n_ − R_0_, R_n_ represents the sensor’s resistance against a particular stimulus, and R_0_ represents its initial resistance.

**Figure 8:**
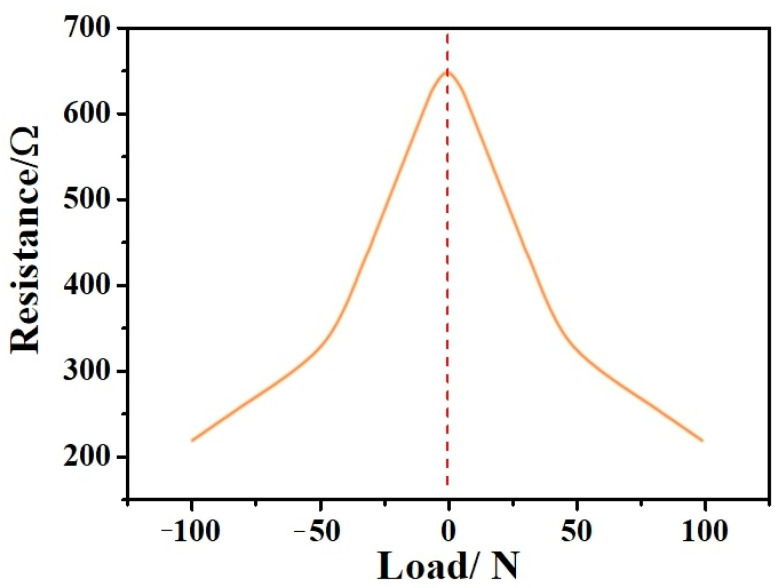
Resistance distribution fitting curve of the sample sponge under one cyclic loading pressure.

Figure 8 shows the resistance distribution fitting curve of the sample sponge under one cyclic loading pressure. As demonstrated by the figure, the left half section of the horizontal axis is force unloading while the right half is force loading. It can be deduced from the results that the maximum value of the resistance fitting curve is when the pressure is equal to 0 N and the sponge porosity is at a maximum. The sponge’s minimum volume is achieved, the pressure is 100 N, and the sponge’s pores are completely closed, equivalent to a conductor with a constant resistance value. Under cyclic loading pressure, the resistance presents a periodic distribution.

**Figure 9:**
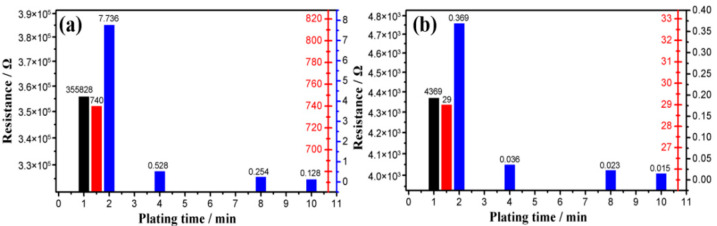
The resistance of sponge with different plating times: (**a**) before compression and (**b**) after compression.

As shown in Figure 9, within the plating time range of 1 to 10 min, the sensor’s conductivity increases with the increasing plating time. The sensor’s conductivity with a 10 min duration is the highest, with its resistance measured at 0.128 Ω under 0 N and 0.015 Ω under 100 N, respectively. In comparison, it is the lowest with a one-minute duration, with its resistance measured at 355,828 Ω at 0 N and 4369 Ω at 100 N. This difference is due to the increase in plating time, the actual coverage of the copper on the unit area of the sensor, and the decrease in the sensor’s equivalent resistance. If the plating time is infinite, theoretically, the copper will completely cover the sponge and fill every pore.

**Figure 10:**
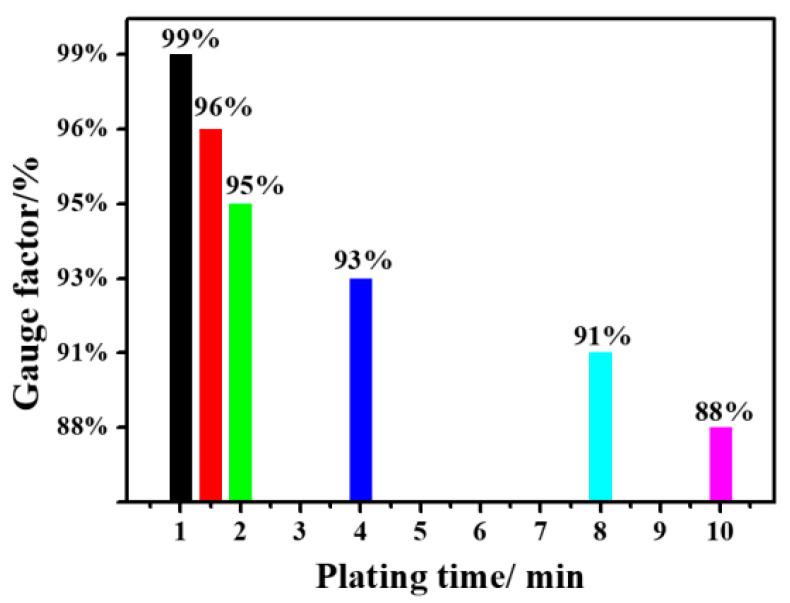
Sponge sensitivity at different plating times.

The piezoresistive sensitivities of sponges with different plating times were compared to determine the sensor with the best performance parameters. The results are shown in Figure 10. In this study, the sensor prepared within one minute of plating time shows the highest sensitivity, at around 99%, while the one prepared with 10 min of plating time has the lowest sensitivity, measured at around 88%. This is due to the porous sponge’s pores being filled with more conductive materials and becoming smaller with the increasing plating time. Therefore, the conductivity is enhanced and the sponge’s resistance to pressure is reduced. It is proven that the sensor’s sensitivity prepared by this process is tunable and has a potential application.

To evaluate the sensor’s performance further, they were compared to the same types of sensors developed in previous literature [47] (as shown in Table 1).

The comparison shows that the sensor developed in this study has higher sensitivity, more stability, and compression recovery properties, and the preparation process is more straightforward and with lower costs compared with others.

### 3.6. Sensor’s Application Analysis

Biocompatibility has to be considered when developing sensors that are usually attached to individuals’ skin and can cause health issues. In this study, copper is attached to the sponge’s pores’ walls. When used for human motion detection, the manufacturing process developed in the study would avoid the possibility that nanoparticles separate from the surface of the elastomer as a result of repeated circulation. It is safe for the user and suitable for commercial human motion detection products. In this study, copper was embedded into the sponge’s pores to significantly reduce the possibility of tearing during use. This advantage makes the sensors developed in this study suitable for wearing.

**Figure 11:**
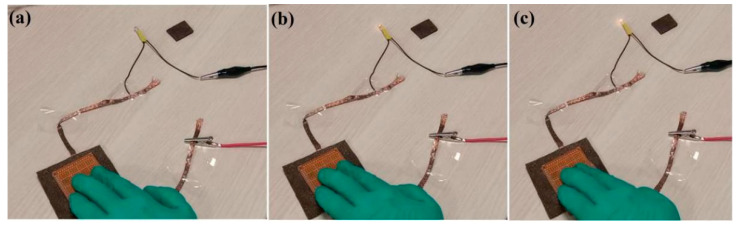
Flexible switch applications: (**a**) unpressed, (**b**) partly pressed, and (**c**) completely pressed.

**Figure 12:**
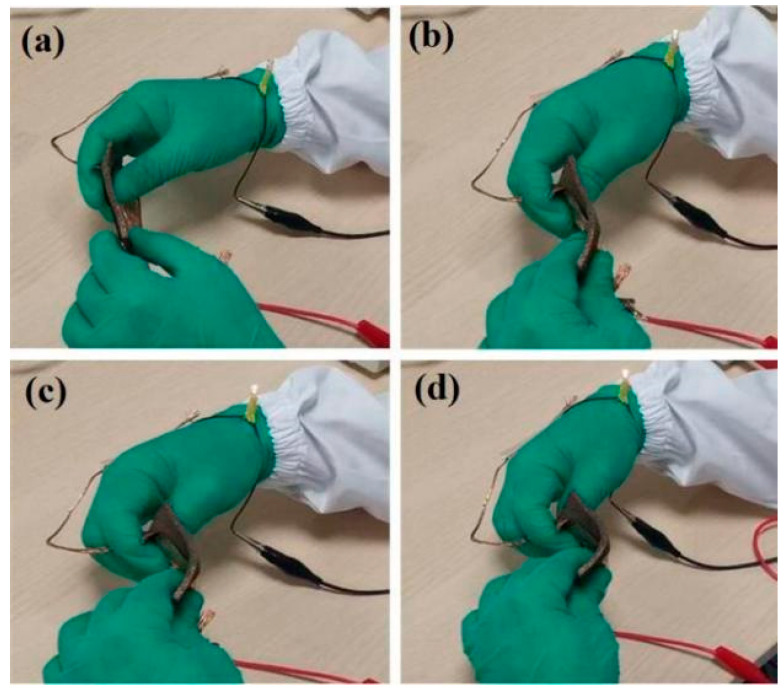
Flexible switch applications: (**a**) 180°, (**b**) 150°, (**c**) 120°, and (**d**) 90°.

In order to visually characterize the sensor’s performance, LED lamps and a power supply were connected to form a series circuit. As shown in Figure 11, the LED lamp is lit when the switch is off, indicating the sensor’s good conductivity. The LED lamp’s brightness is significantly enhanced when pressure is applied to the sensor. Similarly, as shown in Figure 12, by increasing the bending angle, the fitting surface of different shapes is simulated and the LED lamp’s brightness is significantly enhanced. The sensor’s resistance gradually reducing with the compression load can explain this.

The sponge, with its porous structure prepared by electroless plating, has potential applications for monitoring a wide range of pressure stimuli in wearable, flexible electronic gadgets and other related fields.

## 4. Conclusions

Flexible pressure sensors constitute an important part of the applications of flexible electronic sensing systems and have been widely used in biomedical engineering, robotics, and other fields. In the study, a sponge-based flexible sensor with copper as its conductive material is prepared using electroless plating. The sponge’s resistance and sensitivity changes with different plating times are studied. We found that increasing the plating time decreases the resistance value, and sensitivity gradually decreases while the conductivity increases. In this study, the sensor prepared within one minute of plating time shows the highest sensitivity, at around 99%, while the one prepared with 10 min of plating time has the lowest sensitivity, measured at around 88%. Furthermore, the sensor’s applications were assessed, verifying the practicability of the developed preparation method for flexible sensors. This study brings forth new ideas, simplifies the preparation process, and reduces production expenses for flexible pressure sensors.

There are still limitations to this paper, which could be further investigated in future studies: (1) more materials with better conductivity should be considered as sensors’ conductive materials; (2) further improvement in the preparation method and process of the sensors is necessary to advance their overall performance.

## Figures and Tables

**Table 1:** Comparisons of the sensors’ performance.

Materials	Maximum Compression Deformation	Carbon Nano Tube (CNT) Capacity	Carbohydrate Content	Max ∆R/R_0_ (%)
Sponge	50.0%	1.5–3.0%	70.0%	<90.0%
Sponge	50.0%	3.0%	70.0–85.0%	<90.0%
Sponge (this paper)	85.0%	0%	0%	98.8%

## Data Availability

Not applicable.

## References

[1] Trung T.-Q., Lee N.-E. (2016). Flexible and Stretchable Physical Sensor Integrated Platforms for Wearable Human-Activity Monitoring and Personal Healthcare. Adv. Mater..

[2] Mukundan A., Feng S.-W., Weng Y.-H., Tsao Y.-M., Artemkina S.-B., Fedorov V.-E., Lin Y.-S., Huang Y.-C., Wang H.-C. (2022). Optical and Material Characteristics of MoS_2_/Cu_2_O Sensor for Detection of Lung Cancer Cell Types in Hydroplegia. Int. J. Mol. Sci..

[3] Hua Q.-L., Sun J.-L., Liu H.-T., Bao R.-R., Yu R.-M., Zhai J.-Y., Pan C.-F., Wang Z.-L. (2018). Skin-inspired Highly Stretchable and Conformable Matrix Networks for Multifunctional Sensing. Nat. Commun..

[4] Laskowska M., Nowak A., Dulski M., Weigl P., Blochowicz T., Laskowski L. (2021). Spherical Silica Functionalized by 2-Naphthalene Methanol Luminophores as a Phosphorescence Sensor. Int. J. Mol. Sci..

[5] Munteanu I.-G., Apetrei C. (2021). Electrochemical Determination of Chlorogenic Acid in Nutraceuticals Using Voltammetric Sensors Based on Screen-Printed Carbon Electrode Modified with Graphene and Gold Nanoparticles. Int. J. Mol. Sci..

[6] Mannsfeld S., Tee B., Stoltenberg R., Chen C., Barman S., Muir B., Sokolov A., Reese C., Bao Z.-N. (2010). Highly Sensitive Flexible Pressure Sensors with Microstructured Rubber Dielectric Layers. Nat. Mater..

[7] Konishi S., Hirata A. (2019). Flexible Temperature Sensor Integrated with Soft Pneumatic Microactuators for Functional Microfingers. Sci. Rep..

[8] Jeon J., Lee H.-B., Bao Z. (2013). Flexible Wireless Temperature Sensors Based on Ni Microparticle-Filled Binary Polymer Composites. Adv. Mater..

[9] Han Z.-W., Liu L.-P., Zhang J.-Q., Han Q.-G., Wang K.-J., Song H.-L., Wang Z., Jiao Z.-B., Niu S.-C., Ren L.-Q. (2018). High-performance Flexible Strain Sensor with Bio-inspired Crack Arrays. Nanoscale.

[10] Guo H.-Y., Lan C.-Y., Zhou Z.-F., Sun P., Wei D., Li C. (2017). Transparent, Flexible, and Stretchable WS2 Based Humidity Sensors for Electronic Skin. Nanoscale.

[11] Ma L., Wu R., Patil A., Zhu S., Meng Z., Meng H., Hou C., Zhang Y., Liu Q., Yu R. (2019). Full-Textile Wireless Flexible Humidity Sensor for Human Physiological Monitoring. Adv. Funct. Mater..

[12] Huang Y., Fan X.-Y., Chen S.-C., Zhao N. (2019). Emerging Technologies of Flexible Pressure Sensors: Materials, Modeling, Devices, and Manufacturing. Adv. Funct. Mater..

[13] Nela L., Tang J.-S., Cao Q., Tulevski G., Han S.-J. (2018). Large-Area High-Performance Flexible Pressure Sensor with Carbon Nanotube Active Matrix for Electronic Skin. Nano Lett..

[14] Melzer M., Makarov D., Calvimontes A., Karnaushenko D., Baunack S., Kaltofen R., Mei Y., Schmidt O. (2011). Stretchable Magnetoelectronics. Nano Lett..

[15] Chlenova A., Lepalovsky V., Vas’kovskiy V., Svalov A., Kurlyandskaya G. (2017). Magnetoimpedance effect in multilayered permalloy structure with different magnetostriction: Small-pressure sensor. AIP Conf. Proc..

[16] Li B., Kavaldzhiev M., Kosel J. (2015). Flexible Magnetoimpedance Sensor. J. Magn. Magn. Mater..

[17] Yao S., Zhu Y. (2015). Nanomaterial-Enabled Stretchable Conductors: Strategies, Materials and Devices. Adv. Mater..

[18] Zhao S.-F., Li J.-H., Cao D.-X., Zhang G.-P., Li J., Li K., Yang Y., Wang W., Jin Y.-F., Sun R. (2017). Recent Advancements in Flexible and Stretchable Electrodes for Electromechanical Sensors: Strategies, Materials, and Features. ACS Appl. Mater. Interfaces.

[19] Langley D., Giusti G., Mayousse C., Celle C., Bellet D., Simonato J.-P. (2013). Flexible Transparent Conductive Materials Based on Silver Nanowire Networks: A Review. Nanotechnology.

[20] Geim A.-K. (2009). Graphene: Status and prospects. Science.

[21] Kim K.S., Zhao Y., Jang H., Lee S.Y., Kim J.M., Kim K.S., Ahn J.-H., Kim P., Choi J.-Y., Hong B.H. (2009). Large-Scale Pattern Growth of Graphene Films for Stretchable Transparent Electrodes. Nature.

[22] Sekitani T., Nakajima H., Maeda H., Fukushima T., Aida T., Hata K., Someya T. (2009). Stretchable Active-Matrix Organic Light-Emitting Diode Display Using Printable Elastic Conductors. Nat. Mater..

[23] Sekitani T., Noguchi Y., Hata K., Fukushima T., Aida T., Someya T. (2008). A Rubberlike Stretchable Active Matrix Using Elastic Conductors. Science.

[24] Han T., Wang G. (2019). Peroxidase-like Activity of Acetylcholine-Based Colorimetric Detection of Acetylcholinesterase Activity and an Organophosphorus Inhibitor. J. Mater. Chem. B.

[25] Huang W.-P., Li J.-H., Zhao S.-F., Han F., Zhang G.-P., Sun R., Wong C.-P. (2017). Highly Electrically Conductive and Stretchable Copper Nanowires-Based Composite for Flexible and Printable Electronics. Compos. Sci. Technol..

[26] Xu F., Wang X., Zhu Y.-T., Zhu Y. (2012). Wavy Ribbons of Carbon Nanotubes for Stretchable Conductors. Adv. Funct. Mater..

[27] Hu W.-L., Wang R.-R., Lu Y.-F., Pei Q.-B. (2014). An Elastomeric Transparent Composite Electrode Based on Copper Nanowires and Polyurethane. J. Mater. Chem. C.

[28] Lee P., Ham J., Lee J., Hong S., Han S., Suh Y.D., Lee S.E., Yeo J., Lee S.S., Lee D. (2014). Highly Stretchable or Transparent Conductor Fabrication by a Hierarchical Multiscale Hybrid Nanocomposite. Adv. Funct. Mater..

[29] Hu L.-B., Wei Y., Brochu P., Gruner G., Pei Q.-B. (2009). Highly Stretchable, Conductive, and Transparent Nanotube Thin Films. Appl. Phys. Lett..

[30] Zhu Y., Xu F. (2012). Buckling of Aligned Carbon Nanotubes as Stretchable Conductors: A New Manufacturing Strategy. Adv. Mater..

[31] Bowden N., Brittain S., Evans A.-G., Hutchinson J.W., Whitesides G.-M. (1998). Spontaneous Formation of Ordered Structures in Thin Films of Metals Supported on an Elastomeric Polymer. Nature.

[32] Shang Y.-Y., He X.-D., Li Y.-B., Zhang L.-H., Li Z., Ji C.-Y., Shi E.-Z., Li P.-X., Zhu K., Peng Q.-Y. (2012). Super-Stretchable Spring-Like Carbon Nanotube Ropes. Adv. Mater..

[33] Liang H.-W., Guan Q.-F., Zhu Z., Song L.-T., Yao H.-B., Lei X., Yu S.-Y. (2012). Highly Conductive and Stretchable Conductors Fabricated from Bacterial Cellulose. NPG Asia. Mater..

[34] Chen Z.-P., Ren W.-C., Gao L.-B., Liu B.-L., Pei S.-F., Cheng H.-M. (2011). Three-Dimensional Flexible and Conductive Interconnected Graphene Networks Grown by Chemical Vapour Deposition. Nat. Mater..

[35] Yu C.-J., Masampu C., Rong J., Wei B.-Q., Jiang H. (2010). Stretchable Supercapacitors Based on Buckled Single-Walled Carbon-Nanotube Macrofilms. Adv. Mater..

[36] Wu C., Fang L.-J., Huang X.-Y., Jiang P.-K. (2014). Three-Dimensional Highly Conductive Graphene–Silver Nanowire Hybrid Foams for Flexible and Stretchable Conductors. ACS Appl. Mater. Interfaces..

[37] Wu H., Hu L., Rowell M.W., Kong D., Cha J.J., McDonough J.R., Zhu J., Yang Y., McGehee M.D., Cui Y. (2010). Electrospun Metal Nanofiber Webs as High-Performance Transparent Electrode. Nano. Lett..

[38] Zhang Y., Sheehan C.-J., Zhai J., Zou G., Luo H., Xiong J., Zhu Y., Jia Q. (2010). Polymer-Embedded Carbon Nanotube Ribbons for Stretchable Conductors. Adv. Mater..

[39] Chen M., Zhang L., Li C. (2015). Three-Dimensional Porous Stretchable and Conductive Polymer Composites Based on Graphene Network Grown by Chemical Vapour Deposition and PEDOT: PSS Coating. Chem. Commun..

[40] Yu Y., Zeng J.-F., Chen C.-J., Xie Z., Guo R.-S., Liu Z.-L., Zhou X.-C., Yang Y., Zheng Z.-J. (2014). Composite Materials: Three-Dimensional Compressible and Stretchable Conductive Composites. Adv. Mater..

[41] Park J., Wang S., Li M., Ahn C.-G., Hyun J.-K., Dong S.-K., Kim D.-K., Rogers J.-A., Huang Y.-G., Jeon S.-K. (2012). Three-Dimensional Nanonetworks for Giant Stretchability in Dielectrics and Conductors. Nat. Commun..

[42] Chen M.-T., Zhang L., Duan S.-S., Jing S.-L., Jiang H., Li C.-Z. (2015). Highly Stretchable Conductors Integrated with a Conductive Carbon Nanotube/Graphene Network and 3D Porous Poly(dimethylsiloxane). Adv. Funct. Mater..

[43] Wei W., Yang S., Zhou H., Lieberwirth I., Feng X.-L., Müllen K. (2013). 3D Graphene Foams Cross-Linked with Pre-Encapsulated Fe_3_O_4_ Nanospheres for Enhanced Lithium Storage. Adv. Mater..

[44] Xu Y.-X., Sheng K.-X., Li C., Shi G.-Q. (2010). Self-Assembled Graphene Hydrogel via a One-Step Hydrothermal Process. ACS Nano.

[45] Fei H., Li J.-H., Zhao S.-F., Yuan Z., Huang W.-P., Zhang G.-P., Rong S., Wong C.-P. (2017). A Crack-based Nickel@Graphene Wrapped Polyurethane Sponge Ternary Hybrid Obtained by Electrodeposition for Highly Sensitive Wearable Strain Sensors. J. Mater. Chem. C.

[46] Fei H., Su X.-Y., Huang M.-Q., Li J.-H., Zhang Y., Zhao S., Liu F., Zhang B., Wang Y., Zhang G. (2018). Fabrication of a Flexible and Stretchable Three-Dimensional Conductor Based on Au–Ni@Graphene Coated Polyurethane Sponge by Electroless Plating. J. Mater. Chem. C.

[47] Herren B., Webster V., Davidson E., Saha M.-C., Altan M.-C., Liu Y. (2021). PDMS Sponges with Embedded Carbon Nanotubes as Piezoresistive Sensors for Human Motion Detection. Nano Mater..

